# Evaluation of the *In Vitro* Efficacy of *Artemisia annua*, *Rumex abyssinicus*, and *Catha edulis Forsk* Extracts in Cancer and *Trypanosoma brucei* Cells

**DOI:** 10.1155/2013/910308

**Published:** 2013-09-22

**Authors:** Netsanet Worku, Andualem Mossie, August Stich, Arwid Daugschies, Susanne Trettner, Nasr Y. A. Hemdan, Gerd Birkenmeier

**Affiliations:** ^1^Institute of Biochemistry, Faculty of Medicine, University of Leipzig, Johannisallee 30, 04103 Leipzig, Germany; ^2^Institute of Public Health, College of Medicine and Health Sciences, University of Gondar, P.O. Box 198, Gondar, Ethiopia; ^3^Physiology Department, Medical Faculty, Jimma University, P.O. Box 378, Jimma, Ethiopia; ^4^Department of Tropical Medicine, Medical Mission Institute, Salvatorstrasse 7, 97067 Würzburg, Germany; ^5^Department of Veterinary Parasitology, Faculty of Veterinary Medicine, University of Leipzig, An den Tierkliniken 35, 04103 Leipzig, Germany; ^6^Department of Zoology, Faculty of Science, University of Alexandria, Maharram Bey, Alexandria 21511, Egypt

## Abstract

The current drugs against sleeping sickness are derived from cancer chemotherapeutic approaches. Herein, we aimed at evaluating the *in vitro* effect of alcoholic extracts of *Artemisia annua* (AMR), *Rumex abyssinicus* (RMA), and *Catha edulis Forsk* (CEF) on proliferation/viability of 1321N1 astrocytoma, MCF-7 breast cancer, THP-1 leukemia, and LNCaP, Du-145, and PC-3 prostate cancer cells and on *Trypanosoma brucei* cells. Proliferation of tumor cells was evaluated by WST-1 assay and viability/behaviour of *T. brucei* by cell counting and light microscopy. CEF was the most efficient growth inhibitor in comparison to AMR and RMA. Nevertheless, in LNCaP and THP-1 cells, all extracts significantly inhibited tumor growth at 3 *μ*g/mL. All extracts inhibited proliferation of *T. brucei* cells in a concentration-dependent manner. Microscopic analysis revealed that 95% of the *T. brucei* cells died when exposed to 33 *μ*g/mL CEF for 3 hrs. Similar results were obtained using 33 *μ*g/mL AMR for 6 hrs. In case of RMA, however, higher concentrations were necessary to obtain similar effects on *T. brucei*. This demonstrates the antitumor efficacy of these extracts as well as their ability to dampen viability and proliferation of *T. brucei*, suggesting a common mechanism of action on highly proliferative cells, most probably by targeting cell metabolism.

## 1. Introduction 

Many anticancer reagents, including nucleotide analogues and other DNA synthesis inhibitors (e.g., methotrexate), aim at exerting a specific activity against rapidly proliferating cell types. Because of the capacity of *trypanosomes* for rapid growth within mammals, they have been linked to some types of cancer cells. D,L-*α*-Difluoromethylornithine (DFMO), a polyamine synthesis inhibitor, was originally developed as a potential anticancer reagent before it has been proved useful in trypanosomiasis therapy. Furthermore, suramin, melarsoprol, and pentamidine which are drugs licensed for the treatment of human African trypanosomiasis (HAT) are known to arise from cancer research studies [1–3]. If left untreated, sleeping sickness patients die within months when infected with *T. brucei rhodesiense *(acute form of the disease in East and Southern Africa) or within years when infected with *T. brucei gambiense* (chronic form of the disease in West and Central Africa) [4]. It is estimated that at least 300 000 ± 500 000 people are presently infected [5]. Above this burden of the disease, all the available drugs have a number of shortcomings during treatment because of resistance development against them, allergic reactions, undesirable effects in the urinary tract, and reactive encephalopathy (with 3–10% fatality), and some regimens are strict and difficult to apply [6]. This raises an urgent need for novel, safe, rapidly-acting, and inexpensive agents for the treatment of HAT [7].

Our project selects three plants called *Catha edulis Forsk *(*CEF*), *Artemisia annua *(AMR), and *Rumex abyssinicus* (RMA), which are locally endemic to Ethiopia and widely used in the community for various purposes. The pharmacological effect of the selected plants on different cell lines and the known similarity of chemotherapeutics against *trypanosomes* and cancer initiated us to continue investigation of drugs to be commonly effective against these two important diseases.

CEF leaves were commonly used as a stimulant; that was formerly believed to be due to its norpseudoephedrine content. However, it might be cathinone, a newly discovered potent alkaloid with a pharmacological profile closely resembling that of amphetamine, which is responsible to this effect [8]. It can be said that CEF is an amphetamine-like plant material, the effects of which can be accounted to the “khatamines” including phenethylamines cathinone and, to a lesser extent, norpseudoephedrine. In addition to these two “khatamines,” the leaves also contain the cathedulins [8],an interesting group of substances awaiting pharmacological elucidation.

AMR contains artemisinin, an endoperoxide consisting of sesquiterpene lactone [9]. Unlike other peroxide compounds, artemisinin can be stored at room temperature for many years without noticeable decomposition. Semisynthetic derivatives include artemether, arteether, and artesunate [10]. Interestingly, artemisinin is essentially nontoxic to normal cells [11]. Zheng [12] reviewed the cytotoxic activity of artemisinin, and recently it has been shown to be effective in killing cancer cells by inducing apoptosis and inhibition of nuclear factor *κ*-B, an important activator in cancer development and progression [13, 14]. With regard to its antiprotozoal effect, artemisinin has shown a promising antileishmanial activity [15], and currently an artemisinin-based combination therapy (ACT) is recommended for the treatment of *Plasmodium falciparum* malaria [16, 17].

RMA is widely used in folklore medicine for treatment of headache, hemorrhoid, *ascaris*, scabies, leprosy, fungal skin infection, wounds, eczema, and sore-throat and to control mild forms of diabetes. The decoction of the root or leaf powder of the plant is used as vermifuge [18, 19]. Studies have also shown its effect against leukaemia cells by initiating cell death through apoptosis [20]. Unlike the other species of this plant, studies done on the effect of RMA against cancer cells, however, are preliminary.

The objective of this study was, therefore, to evaluate the *in vitro* effect of the three aforementioned plant extracts against cancer cells and *Trypanosoma brucei* cells and to determine the prospect of their common application against these two respective diseases.

## 2. Materials and Methods

### 2.1. Mammalian Cell Culture

Six different tumor cell lines from prostate cancer (PC-3 (CRL-1435, ATCC), Du-145 (ACC 261, DSMZ), and LNCaP (ACC 256, DSMZ)), brain astrocytoma 1321N1 cells (86030102, ECACC); breast cancer MCF-7 cells (ACC 150, DSMZ), and leukemia THP-1 cells (LH-1, ATCC) were cultured in RPMI medium in the absence of fetal calf serum (RPMI-SF) or the presence of 10% fetal calf serum (RPMI-FCS). PC-3 cells, however, were cultured in Dulbecco's modified Eagle's medium in the presence of 10% fetal calf serum (DMEM-FCS). All growing media contain penicillin/streptomycin (100 U penicillin/mL; 100 *μ*g streptomycin/mL), and cells were incubated at 37°C/5% CO_2_. The overall viability of cells used was in the range of 90% to 95%.

### 2.2. *Trypanosoma* Cell Culture

Blood stream forms (BSF) of *T. brucei brucei *laboratory adapted strain (TC-221) cells were cultured in complete Baltz medium, containing 82% Baltz medium basic solution, 0.8% *β*-mercaptoethanol stock solution (20 mM), 0.8% penicillin/streptomycin (10,000 U/mL), and 16.4% heat inactivated FCS all from Biochrom, Berlin, Germany. The 500 mL Baltz medium basic solution contained 3 gm HEPES, 500 mg glucose-monohydrate, 110 mg sodium pyruvate, 7 mg hypoxanthine, 2 mg thymidin, 10.7 mg adenosine, 14.1 mg bathocuproine sulfonate, and 146 mg of L-glutamine, all from Roth (Karlsruhe, Germany) except bathocuproine sulfonate that was purchased from Sigma Aldrich (Seelze, Germany). These compounds were dissolved in 500 mL modified Eagle's medium to which was added 5 mL of nonessential amino acids (Biochrom, Berlin, Germany).

### 2.3. Plant Extracts

Three different medicinal plants (*Catha edulis Forsk*, *Artemisia annua*, and *Rumex abyssinicus*) were deposited in and identified by the National Herbarium, Faculty of Science, Addis Ababa University in Ethiopia. The plants were selected mainly on the basis of their extensive usage in the community and/or on their local medicinal knowledge. The air-dried and powdered plant materials were extracted with 95% methanol at 60°C for 8 hrs using Soxhlet apparatus. The obtained methanol extract was vacuum-filtered and evaporated by using a rotary evaporator and freeze-dried (D. Piatkowski, Munich, Germany). The dried extracts were stored at −20°C until used. Stock solutions were prepared in DMSO just prior to the start of experiments so that its final concentrations did not exceed 1% during the experiment.

### 2.4. Tumor Cells Vitality Test

Proliferation/vitality of tumor cells was assessed using the WST-1 assay according to manufacturer's instructions. This colorimetric assay was used to determine cell proliferation/cytotoxicity in 96 well plates (5000 cells/well). Increasing concentrations of plant extracts (3 to 333 *μ*g/mL) were added after preincubation of cells for 24 hrs (start value). A volume of 12 *μ*L stock solution of WST-1 was added to each well plate, and was incubated for 4 hrs at 37°C. As a control, DMSO was added instead of the plant extracts. The absorbance was directly recorded on an ELISA reader/96-well multiscanner at a wavelength of 450 nm.

### 2.5. Impact of Exposure of Plant Extracts on Survival of *Trypanosoma brucei* Cells

Time dependency test was done in 24 well plates where 1 mL of cell suspension (2 × 10^5^ cells) plus 100 *μ*L of plant extracts giving a total volume of 1.1 mL/well were incubated for 24 hrs at 37°C in humidified environment containing 5% CO_2_. To count the cells first the plate was slowly shaken in circular motion and well mixed using a 100 *μ*L pipette to help the cells float well in the media just before pipetting for counting on a Neubauer hemacytometer. Ten microliters of the cell suspension has been taken, and the number of moving cells was then recorded after 1 hr, 3 hrs, 6 hrs, and 24 hrs of incubation. Tests have been performed in triplicates, and the average values were obtained for two repeated experiments (*n* = 6). The controls were seeded in a 1.1 mL of fresh medium containing 1% DMSO and handled in exactly the same way as the exposed cells.

### 2.6. Microscopic Observation of* Trypanosoma brucei* Cells' Viability

This test was done using 24 well plates containing 1 mL of cell suspension (2 × 10^5^ cells) plus 100 *μ*L of plant extract. Cells were exposed to three different concentrations of the respective plant extracts, and the activity of cells was evaluated by monitoring (i) movement of live cells, (ii) morphology of the moving and nonmoving cells, and (iii) the number of live moving cells recorded at the 2nd, 3rd, 6th, and 24th hr of incubation at 37°C in humidified environment containing 5% CO_2_ and compared with the controls.

### 2.7. Statistical Analysis

Count and percentage of dead/viable cells has been estimated to compare effect of plant extracts on exposed cells and the corresponding controls. The optical density reading was used to determine the relative rate of cell proliferation to determine the IC_50_ of plant extracts using a linear interpolation. The statistical significance of difference of two means in cancer cells experiment was calculated using unpaired *t*-test, and values of *P* < 0.05 were considered to be statistically significant. The mean and standard deviation values are also employed for comparison. The data analysis, all figures, and tables presented were done using GraphPad Prism (version 4) statistical software.

## 3. Results

### 3.1. *In Vitro* Evaluation of Anticancer Effect

**Figure 1:**
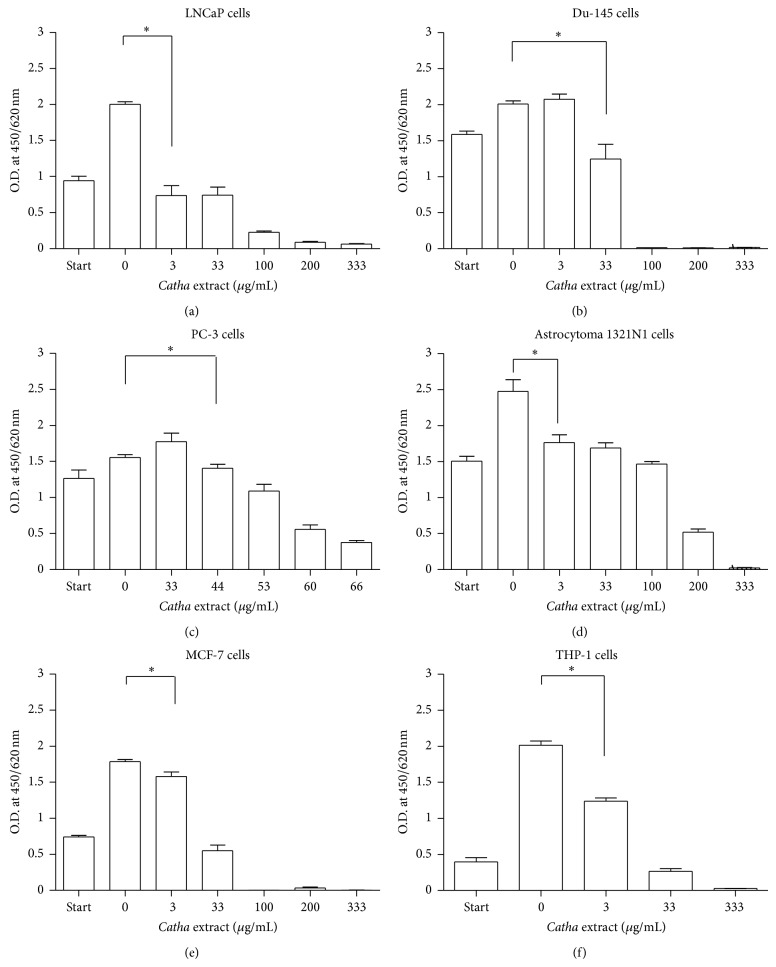
Inhibition of cancer cell growth by *Catha edulis Forsk *(CEF) extract. A colorimetric assay was used to determine proliferation of tumour cells (5000 cells/well in a 96 well plate) exposed to different concentrations of CEF extract (3–333 *μ*g/mL) using a 33 mg/mL stock solution. WST-1 was added to each well plate and incubated for 4 hrs at 37°C. As a control, DMSO was added instead of the plant extract (zero value). The absorbance was measured at a wavelength of 450/620 nm by an ELISA reader. The reduction in cell number was calculated from the absorbance given in percent. Statistical significance was calculated using unpaired *t*-test, at 95% CI (∗).

CEF showed concentration-dependent inhibitory effect in all cancer cells tested, and a statistically significant inhibition of proliferation (*P* < 0.05) was observed at 3 *μ*g/mL concentration in LNCaP, astrocytoma 1321N1, MCF-7, and THP-1 cells. When compared with the other cancer cell types tested, the lowest recorded IC_50_ was found in case of LNCaP prostate cancer cells (2.4 *μ*g/mL). In addition to LNCaP prostate cancer cells, CEF best inhibits THP-1 leukemia and MCF-7 cells when applied at concentrations ≥33 *μ*g/mL. In all cases, the maximum concentration applied resulted in nearly 100% inhibition. In PC-3 cells, however, maximum constant inhibition has been reached at 66 *μ*g/mL (Figure 1).

**Figure 2:**
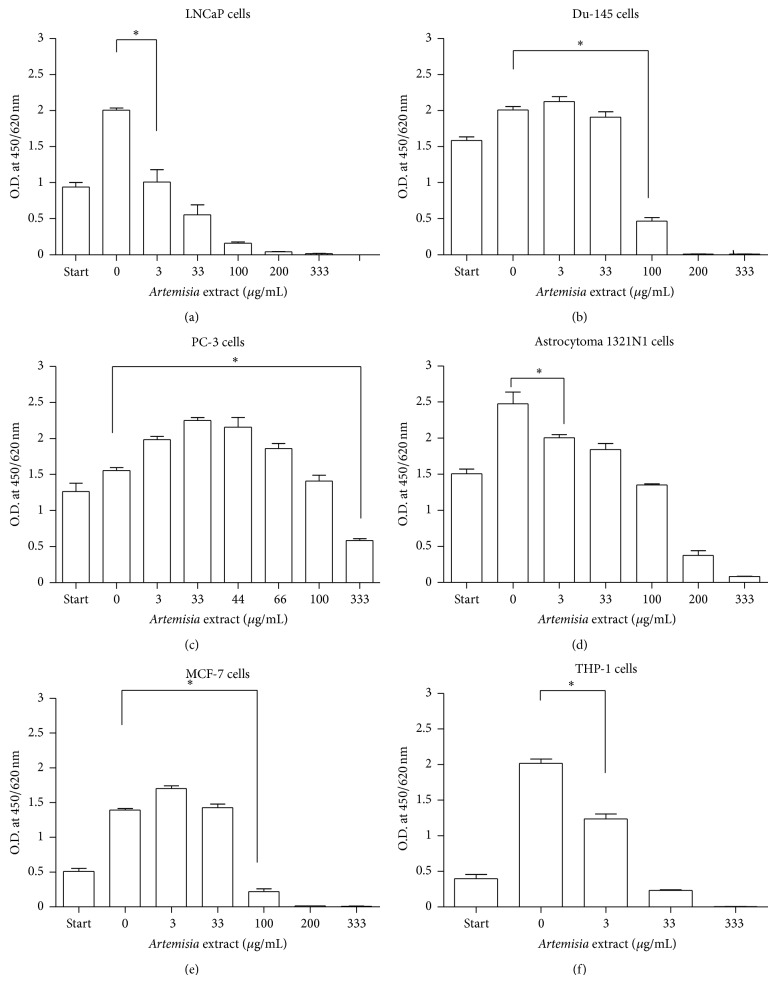
Inhibition of cancer cell growth by *Artemisia annua* (AMR) extract. A colorimetric assay was used to determine proliferation of tumour cells (5000 cells/well in a 96 well plate) exposed to different concentrations of AMR extract (3–333 *μ*g/mL). After incubation periods of 24 hrs, 12 *μ*L stock solution of WST-1 was added to each well plate and incubated for 4 hrs at 37°C. As a control, DMSO was added instead of the plant extract (zero value). The absorbance was measured at a wavelength of 450/620 nm by an ELISA reader. The reduction in cell number was calculated from the absorbance given in percent. Statistical significance was calculated using unpaired *t*-test, at 95% CI (∗).

AMR exerts a concentration-dependent effect on all cancer cells undertested except in PC-3 cells in which case it looks to have a provital effect at lower concentrations ≤33 *μ*g/mL (Figure 2). In LNCaP, 1321N1, and THP-1 cells it revealed a statistically significant inhibition (*P* < 0.05) at a concentration starting from 3 *μ*g/mL. In the remaining three cancer cell types, however, a significant inhibition has reached concentrations greater than or equal to 100 *μ*g/mL. The lowest IC_50_ (3 *μ*g/mL) has been recorded in LNCaP cells when compared with the remaining cancer cells employed in our experimental study (Figure 2). 

**Figure 3:**
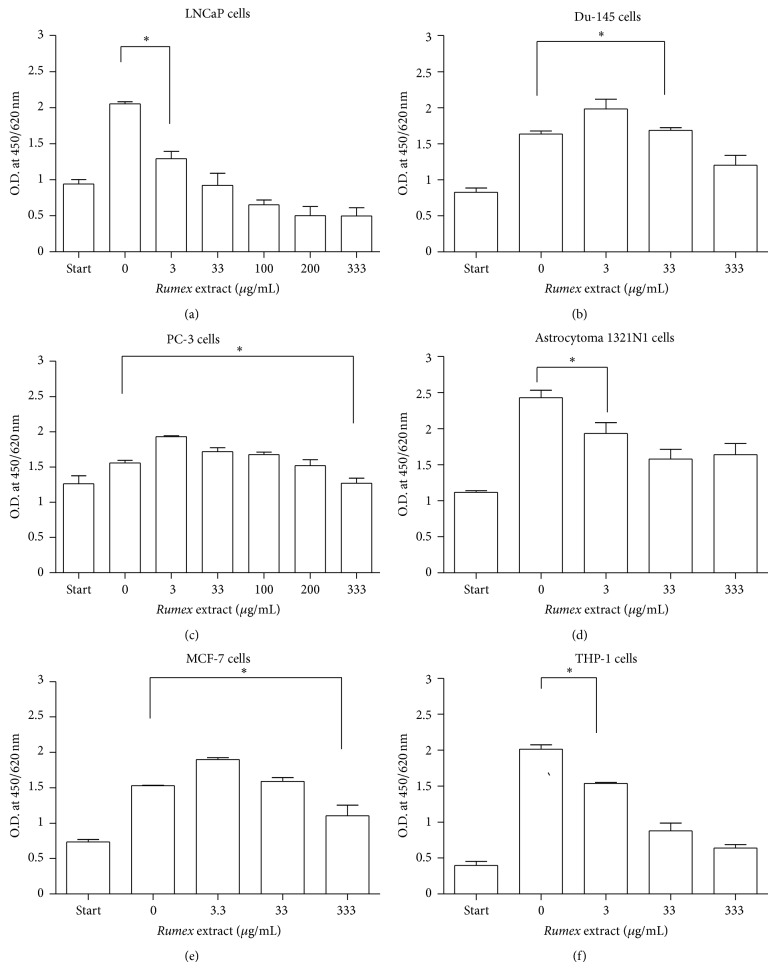
Inhibition of cancer cell growth by *Rumex abyssinicus* (RMA) extract. A colorimetric assay was used to determine proliferation of tumour cells (5000 cells/well in a 96 well plate) exposed to different concentrations of RMA extract (3–333 *μ*g/mL). After incubation periods of 24 hrs, 12 *μ*L stock solution of WST-1 was added to each well plate and incubated for 4 hrs at 37°C. As a control, DMSO was added instead of the plant extract (zero value). The absorbance was measured at a wavelength of 450/620 nm by an ELISA reader. The reduction in cell number was calculated from the absorbance given in percent. Statistical significance was calculated using unpaired *t*-test, at 95% CI (∗).

In case of RMA, the concentration-dependent inhibitory effect has also been well documented against growth of all cancer cell lines. A statistically significant growth inhibition (*P* < 0.05) was observed at 3 *μ*g/mL in LNCaP, 1321N1, and THP-1 cells. Unlike the other two plant extracts, however, the degree of growth inhibition by *Rumex abyssinicus* extract was low as it has not resulted in a complete inhibition of multiplication even at the maximum concentration (333 *μ*g/mL) in all cancer cells tested. The lowest IC_50_ (29 *μ*g/mL) was recorded in THP-1 cells. In these cells the maximum dilution had resulted in only 70% inhibition (Figure 3).

### 3.2. *In Vitro* Evaluation of Antitrypanosomal Effect

The proliferation of *T. brucei* cells has been assayed using Alamar blue, an indicator for metabolic cell function, evaluated as a colorimetric dye. However, when the Alamar blue was mixed with the growth media, the deep red or yellow coloration of the extracts at higher concentrations interferes with the absorbance measurement. For this reason, we have employed the time dependency test by counting the number of actively moving cells after exposing the cells to the plant extracts for 24 hrs at 37°C in 24 well plates. Cell counting has been done at the start of seeding, and at 1 hr, 3 hrs, 6 hrs and 24 hrs incubation periods. Microscopic observation on the relative speed of cellular motility and their morphological changes has also been recorded.

**Figure 4:**
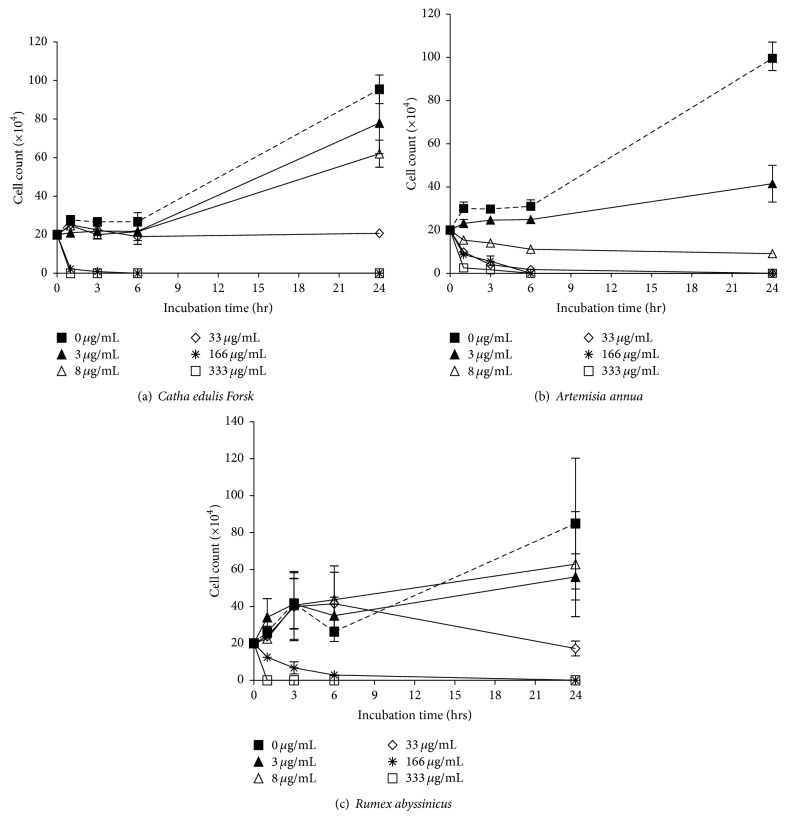
Exposure of *Trypanosoma brucei* cell to (a) *Catha edulis Forsk *(CEF), (b) *Artemisia annua* (AMR), and (c) *Rumex abyssinicus *(RMA) extracts. Cells (2 × 10^5^ cells per well) were seeded in 24 well plates in a volume of 1 mL per well, and 100 *μ*L of extracts was inoculated in each well except in the controls where 100 *μ*L of media is inoculated instead of the extracts. The cells were incubated at 37°C containing 5% CO_2_ in a 100% humidified environment for 24 hrs. Cell counts were made at 0, 1, 3, 6, and 24 hrs of incubation.

Results demonstrated that CEF inhibited proliferation of *T. brucei *cells at the last two higher dilutions (166 and 333 *μ*g/mL), and its initial effect was observed within the first hour of incubation, and its maximum effect was seen at the 6th hr (Figure 4(a) and Table 1). At concentrations of 3 and 8 *μ*g/mL, no inhibitory effect was observed. At 33 *μ*g/mL, however, cytostatic effect was observed where the number of cells remains almost constant within the following 24 hrs of incubation (Figure 4(a)). 

AMR similarly inhibits growth of cells at concentrations of 33 *μ*g/mL, 166 *μ*g/mL, and 333 *μ*g/mL. The inhibitory effect was detected following the first hour of incubation and continues until it kills all the cells within the next 24 hrs of incubation (Figure 4(b) and Table 1). At 3 *μ*g/mL and 8 *μ*g/mL concentrations, however, no inhibition had been observed whereby it resulted in a cytostatic effect at 8 *μ*g/mL, as the number of cells kept constant over a time elapse of 24 hrs (Figure 4(b)). 

RMA also results in 100% inhibition at ≥166 *μ*g/mL after 24 hrs of incubation. At 33 *μ*g/mL, the cell number had decreased to a value of about 30%, but no inhibitory effect at 3 or 8 *μ*g/mL was observed (Figure 4(c)). Table 1 lists the phenotypic changes recorded upon monitoring cells exposed to the various concentrations of CEF, AMR, and RMA extracts.

## 4. Discussion

Although cancer is not a single disease, and *Trypanosoma* are eukaryotic parasites presenting a high level of genotypic variability, the growth of the parasites in their host and that of cancer cells share at least one common feature, that is, their mutual capacity for rapid cell division [21]. Such cells require high polyamine concentration, and the inhibition of polyamine metabolism highly affects their survival. For example, the drug eflornithine (DFMO) is known to affect the polyamine synthesis in both *Trypanosoma* and cancer cells. 

Another area of interest linking these two cell types is the glycolytic pathway. Tumor cells gain their energy mainly from aerobic glycolysis, and *trypanosomes* solely depend on glycolysis which is critical to their survival. For example, silencing the hexokinase 1 (*Tb*HK1) gene in *T. brucei* induced parasite death, and a similar effect has been observed using the hexokinase inhibitor lonidamine, both in cancer and trypanosome cells [22].

For the treatment of sleeping sickness only four drugs, of which three were developed 50 years earlier, are available. Eflornithine (DFMO), a selective inhibitor of ornithine DL-decarboxylase, is the only new drug for chemotherapy of sleeping sickness. It was first used in 1990 and is only effective against *T. b. gambiense *[23]. With the exception of the prodrug pafuramidine maleate (DB289), an orally bioavailable diamidine about to enter Phase II clinical trials [24], no other compounds are in clinical development, indicating that no new antisleeping sickness drug will become available in the next few years [25]. In the light of declining investment in drug development for parasitic diseases, new strategies for the chemotherapy of HAT are urgently required [26]. Among these strategies it is promising to employ drugs developed as potential antitumour agents in the fight against HAT. The current antitrypanosoma drugs including suramin, pentamidine, melarsoprol, and eflornithine are good examples supporting this strategy [23, 25–27]. Similarly, (−)-epigallocatechin-3-gallate, a constituent of green tea, has antioxidant, anti-inflammatory, antimicrobial, anticancer, and antitrypanocidal activities [28]. Investigation of other plants with antitrypanosoma and anticancer activities is the easiest way to evade the long and costly process of development of new antisleeping sickness drugs. Here we, therefore, present the pharmacological activity of plants *Artemisia annua*, *Rumex abyssinicus*, and *Catha edulis Forsk* against *T. brucei* and different cancer cells.

By comparing the efficacy of these three plant extracts in inhibition of proliferation of cancer cell lines, we have observed that CEF was more effective than AMR and RMA, showing a statistically significant inhibition in four of the cancer cell types at the lowest concentration used (3 *μ*g/mL). On the other hand, when the complete inhibitory effect of the maximum dilution was considered, RMA was not as effective as AMR and CEF. Among all cell lines tested, LNCaP prostate cancer and THP-1 leukemia cells were found to be most susceptible to the inhibitory effects of all the three plant extracts, where their IC_50_s lay below 33 *μ*g/mL. In addition, it was observed that extracts of CEF, AMR, and RMA displayed antiproliferative effects on *T. brucei* cells. Best inhibitory effects were observed at concentrations ranging between 33 *μ*g/mL and 333 *μ*g/mL. Similarly, *T. brucei* cells proved to be most susceptible to CEF and AMR (Figure 4 and Table 1). As it was observed in a microscope, the effect of the latter two extracts was detected after 2 hours of incubation at concentrations ≥33 *μ*g/mL (Table 1). Overall, all the three plant extracts exerted their inhibitory effect, even though with variable efficacies, both in cancer and trypanosome cells. This result is in accordance with previous findings using various cancer cell lines and trypanosome species [8, 29–31].

Our study on *T. brucei* cells has demonstrated that 98% of cells died after 6 hrs at 33 *μ*g/mL of the CEF extract (Table 1). A study done by Dimba et al. [32] described a novel mechanism of CEF-induced cell death by apoptosis involving induction of caspases in HL-60 leukemia cell lines. They had also shown that its effect is rapid resulting in cell death within 2 hrs. A similar study done by Rosenkranz and Wink [33] has also shown that alkaloids induce apoptosis in blood stream form *T. brucei*. Alkaloids are known to affect protein biosynthesis, interact with DNA, and disturb membrane fluidity and/or microtubule formation. This might be the reason for the rapid cell death observed in *T. brucei* cells in our study. However, the type of cell death process in our experiments must be clearly demonstrated in future studies.

The current experimental study similarly had shown a good inhibitory effect of CEF on THP-1 leukemia cells, starting from 33 *μ*g/mL, which is in agreement with the study done by Dimba et al. [32]. In the other cancer cell lines used in the current study, CEF extract efficiently inhibited cell proliferation even at the lower concentration, supporting its activity as an anticancer and antitrypanosomal drug. This result, therefore, is in agreement with *in vitro* antimicrobial property studies against periodontal pathogens done by Al-Hebshi et al. [34]. 

As reviewed by Sinclair et al. [35] on the pharmacological effect of artemisinin on cancer cells, it is indicated that its mechanism of action is done by affecting the transferrin receptor in iron metabolism that is pertinent for survival in almost all cancer cells. Transferrin receptors are highly expressed in cell surfaces of breast cancer, brain tumour glioblastoma and meningo-glioma as well as in myelogenous leukaemia (CML) cells compared to their corresponding normal cells. Leukaemia cells were found to be most sensitive to artesunate. Accordingly, our result shown in Figure 2 was partly in support of these findings whereby a significant cell growth inhibition was observed both in 1321N1 and THP-1 cells at the lowest applied concentration. The MCF-7 cells were also affected significantly, but at a higher concentration than in case of the previous two cell lines. This warrants further investigation to identify the reason for their lower susceptibility albeit the high expression of transferrin receptors on their surfaces.

Artemisinin contains an endoperoxide bridge (R–O–O–R′) that interacts with Fe (II) to form toxic free radicals and is required for its antimalarial as well as anticancer activity. It is believed to exert its cytotoxic effect via Fe (II)-mediated cleavage of the endoperoxide bridge [11, 36]. An intact endoperoxide is crucial since artemisinin derivatives lacking an endoperoxide bridge are devoid of antimalarial activity [17]. In the case of bloodstream forms of *T. brucei*, iron is delivered by host transferrin to a heterodimeric, glycosylphosphatidylinositol-anchored transferrin receptor [37]. The trypanosomal transferrin receptor is homologous to the N-terminal domain of the variant surface glycoprotein (VSG) and bears no structural similarity with the human transferrin receptor [37]. This structural difference to the human transferrin receptor makes this pathway a good target to develop chemotherapy against human trypanosomiasis. A study done by Mishina et al. [38] also confirmed the inhibitory effect of artemisinin in the *in vitro* growth of *T. cruzi* and *T. b. rhodesiense* in low micromolar range. It targets the calcium dependent-ATPase activity in *T. cruzi* cell membrane. With respect to this fact, the strong efficacy of AMR to inhibit *T. brucei* cells' proliferation revealed during the current study is quite appreciated, where nearly 50% of cells died within 3 hrs of incubation (Table 1) and 100% after 24 hrs of incubation at 33 *μ*g/mL (Figure 4(b)).

An *in vitro* experiment done by Maksimović et al. [39] on the fruit of the yellow dock *Rumex crispus* indicated that it has a specific antioxidant effect. It has also been identified that ethyl acetate extracts from the roots of *Rumex dentatus* contained four spectroscopically separable compounds and two of which were novel. From these four identified compounds chrysophanol exerted a better inhibition of growth in MCF-7 cell lines [40]. In the current study, *Rumex abyssinicus* selectively showed better inhibition in THP-1 and LNCaP cells that corresponds with the study done by Wagiera et al. [20] using *Rumex dentatus*. However, our findings did not agree with regard to the effect of this plant extract on MCF-7 cells when compared with the study done by Zhang et al. [40]. Otherwise, our study revealed that methanol extract of *Rumex abyssinicus* was not a good candidate of therapy mainly against MCF-7 and PC-3 cells, where by a statistically significant inhibition (*P* < 0.05) has been seen only at the maximum concentration applied (333 *μ*g/mL).

## 5. Conclusions

Our present study demonstrates the efficacy of CEF, AMR, and RMA in inhibiting growth of different cancer cell lines as well as *T. brucei* cells. The CEF extract was relatively the best effective extract in comparison to AMR and RMA against proliferation of all cell lines. In all cases, LNCaP and THP-1 cells were found to be the most susceptible cell lines among others. It has been recalled that almost all of the drugs against human African trypanosomiasis are derived from drugs for cancer. Our current results will also strengthen this consent and can be applied towards developing drugs to be commonly used in sleeping sickness and cancer. This area of integrated “cancer and neglected tropical diseases (NTDs)” research should further be promoted so that NTDs management suffering from various problems like drug resistance, insecticide resistance of transmitting vectors, and financial shortage of production of new drugs can probably be solved.

## Figures and Tables

**Table 1:** Efficacy of alcoholic extracts of *Rumex abyssinicus* (RMA), *Artemisia annua* (AMR), and *Catha edulis Forsk* (CEF) on *Trypanosoma* cells. *T. brucei* cells were seeded in 96 well plates (2 × 10^4^ cells/well), and extracts of the different plants were added in increasing concentrations. The cells were inspected at the 2nd, 3rd, 6th, and 24th hrs of incubation by microscopic observation according to the following operational definitions: live cells: cells moving either fast or slowly; dead cells: no moving cells are observed in the medium; actively moving: cells move actively swimming from place to place in the medium within the microscopic field; weakly moving: a live cell that moves slowly in a fixed position; clamping: cells sticking together in groups of a minimum of 5 cells per clamp.

Concentration (µg/mL)	RMA	AMR	CEF
	2 hr incubation		
3	XXXXX	XXXXX	XXXXX
33	XXXXX	XXXX	XXX
333	X	XX	X

	3 hr incubation		
3	XXXXX	XXXXX	XXXXX
33	XXXXX	XXXX	XX
333	X	X	X

	6 hr incubation		
3	XXXXX	XXXXX	XXXXX
33	XXXXX	XX	XX
333	X	X	X

	24 hr incubation		
3	XXXXX	XXXXX	XXX
33	XXXXX	X	X
333	X	X	X

X: all cells are dead; XX: more than 95% of cells are dead, and the rest are slowly moving at a fixed position; XXX: all cells move weakly and start clamping; XXXX: 50% of cells are active, and 50% are weakly moving; XXXXX: live and actively moving cells.
